# NLRP7 is expressed in the ovine ovary and associated with *in vitro* pre-implantation embryo development

**DOI:** 10.1530/REP-19-0081

**Published:** 2019-09-10

**Authors:** Guangdong Li, Xiuzhi Tian, Dongying Lv, Lu Zhang, Zhenzhen Zhang, Jing Wang, Minghui Yang, Jingli Tao, Teng Ma, Hao Wu, Pengyun Ji, Yingjie Wu, Zhengxing Lian, Wei Cui, Guoshi Liu

**Affiliations:** 1 Beijing Key Laboratory for Animal Genetic Improvement, National Engineering Laboratory for Animal Breeding, Key Laboratory of Animal Genetics and Breeding of the Ministry of Agriculture, College of Animal Science and Technology, China Agricultural University, Beijing, China; 2 Institute of Animal Sciences, Chinese Academy of Agricultural Sciences, Beijing, China; 3 Department of Surgery and Cancer, Institute of Reproductive and Developmental Biology, Imperial College London, London, UK; 4 Beijing Advanced Innovation Center for Food Nutrition and Human Health, China Agricultural University, Beijing, China

## Abstract

*NLRP* (NACHT, LRR and PYD domain-containing proteins) family plays pivotal roles in mammalian reproduction. Mutation of *NLRP7* is often associated with human recurrent hydatidiform moles. Few studies regarding the functions of *NLRP7* have been performed in other mammalian species rather than humans. In the current study, for the first time, the function of *NLRP7* has been explored in ovine ovary. NLRP7 protein was mainly located in ovarian follicles and in *in vitro* pre-implantation embryos. To identify its origin, 763 bp partial CDS of *NLRP7* deriving from sheep cumulus oocyte complexes (COCs) was cloned, it showed a great homology with *Homo sapiens*. The high levels of mRNA and protein of NLRP7 were steadily expressed in oocytes, parthenogenetic embryos or IVF embryos. *NLRP7* knockdown by the combination of siRNA and shRNA jeopardized both the parthenogenetic and IVF embryo development. These results strongly suggest that *NLRP7* plays an important role in ovine reproduction. The potential mechanisms of *NLRP7* will be fully investigated in the future.

## Introduction

NLRs (nucleotide-binding and oligomerization domain (NOD)-like receptors), also called CATERPILLER (caspase recruitment domain, transcription enhancer, purine binding, pyrin, lots of leucine repeats), serve as the intracellular guards to coordinate the innate immunity and inflammatory responses after perception of adverse signals within the cell (Barbé *et al.* 2014, Meunier & Broz 2017). Recently, NLRs have also emerged as the key regulators of folliculogenesis and early embryonic development in mammals. A subset of phylogenetically related NLRs represents a new category of maternal genes which are highly expressed in oocytes and pre-implantation embryos. Mutations of these genes might lead to hereditary reproductive defects and imprinting diseases (Van Gorp *et al.* 2014). Four subfamilies of NLRs are classified based on different N-terminal effector domain: NLRA, NLRB, NLRC and NLRP. For example, the *NLRP* family contains a pyrin domain (PYD) (Ting *et al.* 2008). Fourteen members of *NLRP* family are found in *Macaca mulatta* and *Homo sapiens* and 20 members are present in *Mus musculus* (Zhang *et al.* 2008, McDaniel & Wu 2009). In the process of evolution, *NLRP1*, *4*, *9* have duplicated, while *NLRP7*, *8*, *11* and *13* have been lost. Interestingly, in humans, nine NLRP proteins (NLRP2, 4, 5, 7, 8, 9, 11, 13 and 14) are reproduction related and play the crucial roles in the reproductive system (Tian *et al.* 2009).

*NLRP7*, also known as *NALP7* (neuronal apoptosis LRR pyrin domain protein 7), *NOD12* (nucleotide-binding oligomerization domain protein 12), *PYPAF3* (PYRIN-containing APAF1-like protein 3), *CLR19.4*, *PAN7* and *HYDM*, belongs to the NLRP family and locates into human 19q13.4. It plays multiple roles including immunity and reproduction (Van Gorp *et al.* 2014). The structure of NLRP7 has a central large NACHT (NAIP, CIITA, HET-E, TP1) domain with a nuclear localization signal, an N-terminal PYD (pyrin) domain involving protein–protein interactions and downstream signal pathways and an LRR (leucine-rich repeats) domain that varies in length depending on splicing isoforms (Slim & Wallace 2013, Reddy *et al.* 2016). *NLRP7* has no ortholog in the rodents, but has a paralog, *NLRP2*. *NLRP7* probably emerged from *NLRP2* by gene duplication during evolution (Duéñez-Guzmán & Haig 2014). In the mouse oocytes, *NLRP2* knockdown leads to embryonic arrest between two- and eight-cell stages (Peng *et al.* 2012).

Previously, *NLRP7* was described as an inhibitor of the inflammasome signal pathway, its overexpression in HEK-293T cells impaired *caspase-1*-mediated *IL-1β* production (Kinoshita *et al.* 2005). In contrast, recent evidence suggested that *NLRP7* induced an inflammasome formation in response to microbial acylated lipopeptides and promoted inflammatory cytokines production (Khare *et al.* 2012, Radian *et al.* 2013,^ 2015^, Zhou *et al.* 2016). In peripheral blood mononuclear cells, *NLRP7* localizes to the microtubule-organizing center, the Golgi apparatus and associates with microtubules. This suggests that it may coordinate cytokines secretion and transportation (Messaed *et al.* 2011). In addition, *NLRP7* is referred to as a maternal-effect gene, whose mutations commonly result in recurrent hydatidiform moles (RHMs), a gestational trophoblastic disease characterized by a mass exhibiting trophoblastic hyperplasia and swelling of chorionic villi as well as impaired embryonic development (Murdoch *et al.* 2006, Sebire *et al.* 2013, Nguyen *et al.* 2014, Carey *et al.* 2015, Ito *et al.* 2016, Sills *et al.* 2017, Kalogiannidis *et al.* 2018). The homozygous or compound heterozygous *NLRP7* missense and non-sense mutations in male do not jeopardize their normal reproductive outcomes and this indicates that *NLRP7* may specifically regulate female reproduction (Qian *et al.* 2007, Wang *et al.* 2009). Thus, excepting for an inflammatory response, *NLRP7* may have another important role related to female reproduction, that is, it is present in the oocyte paralleling other maternal-effect genes to regulate female reproductive activities. For example, ovum donation has rescued defects in patients with recessive mutations in *NLRP7* (Fisher *et al.* 2011, Nguyen *et al.* 2014, Akoury *et al.* 2015). It is still controversial whether mutations in *NLRP7* also contribute to the etiology of other forms of molar pregnancies and reproductive wastage syndromes (Slim *et al.* 2011, Andreasen *et al.* 2012, Brown *et al.* 2013, Manokhina *et al.* 2013, Slim & Wallace 2013, Mahadevan *et al.* 2014). As yet, the exact mechanisms of *NLRP7* in imprinting defects on abnormal pregnancies are still in debate (Sanchez-Delgado *et al.* 2015, Singer *et al.* 2015, Soellner *et al.* 2017, Reynaud *et al.* 2018) and are complicated by the variety of disease phenotypes identified.

Majority of the studies on *NLRP7* were focused on its effects on human recurrent hydatidiform moles and a few on other animals. Thus, *NLRP7* was referred to as a primate-specific *NLR* (Van Gorp *et al.* 2014). Until now, there was no report on the expression, localization, and function of *NLRP7* in the ovine species. Therefore, the main purpose of this study was to elucidate the potential roles of *NLRP7* in the non-primates, that is, sheep.

## Materials and methods

### Chemicals

Unless otherwise stated, reagents were purchased from Sigma Chemical Co..

### Animal studies and ethics statement

All experimental procedures concerning the handling of sheep strictly followed protocols approved by the Animal Welfare Committee of China Agricultural University (Permit Number: SYXK2015002), and this study was carried out in strict accordance with the guidelines and regulations established by this committee.

### Sheep tissues collection

The tissues used in this study were taken from slaughterhouse in the Dachang Hui Autonomous County in Langfang City, Hebei Province, China. Heart, liver, spleen, kidney, stomach (rumen), uterus, oviduct, testis (head of epididymis), ovary, placenta (from ewes with 3 months of pregnancy), hypothalamus, pituitary, pineal gland and corpus luteum were randomly sampled from three individual adult (4–6 years old) small-tailed Han sheep (*Ovis aries*). Tissues were quickly frozen in liquid nitrogen and stored at −80°C until RNA extraction.

### RT-PCR and quantitative real-time PCR

Total RNA was isolated from adult ovine tissues using TRIzol Reagent (Invitrogen) according to the manufacturer’s protocol and the total RNA concentration was quantified using a NanoDrop 2000 Spectrophotometer (Thermo Fisher Scientific). cDNA synthesis was conducted using PrimeScript II 1st Strand cDNA Synthesis Kit (Takara) with Oligo dT Primers (50 µM) and Random 6 mers (50 µM).

The primer sequences used for RT-PCR and quantitative real-time PCR were listed in Table 1. cDNA samples were amplified by RT-PCR with Ex Taq DNA polymerase (Takara). The cycling parameters were an initial denaturation of 2 min, 95°C, followed by 30 cycles of 94°C for 30 s, 60°C for 35 s, 72°C for 40 s and a final extension at 72°C for 10 min. The amplified products were evaluated by a 1.5% agarose gel containing ethidium bromide. After purification, the PCR products were sequenced and sequence identities were confirmed using BLAST (http://www.ncbi.nlm.nih.gov/BLAST/). The mRNA abundance was quantified on the LightCycler 480 II instrument (Roche) using LightCycler® 480 SYBR Green I Master (Roche). The thermal program included a 10-min incubation at 95°C to activate FastStart DNA polymerase, followed by 35 cycles of 95°C for 10 s, 60°C for 15 s and 72°C for the appropriate extension time with single fluorescence acquisition.

**Table 1:** Primer sets for relative quantification of *NLRP7*/*β-actin*/*GAPDH* genes.

Gene	Accession number	Forward primer	Reverse primer	Tm (°C)	Length (bp)
*NLRP7*	XM_004015893.4	5′-TGCTTACCGGGACTTCTGTC-3′	5′-CCACTGCCAAGTGGTGTCA-3′	60	181 (spanning the third intron)
*β-actin*	NM_001009784.3	5′-CTCTTCCAGCCTTCCTTCCT-3′	5′-GGGCAGTGATCTCTTTCTGC-3′	58	178 (spanning the fourth intron)
*GAPDH*	XM_027961471.1	5′-CTGGCCAAGGTCATCCAT-3′	5′-ACAGTCTTCTGGGTGGCAGT-3′	60	86 (spanning the seventh intron)

Comparisons among several tissues should be performed with at least two endogenous references (MiQE guidelines: http://www.rdml.org/miqe.php). The relative expression was normalized by the corresponding geometric average of the two housekeeping genes (reference genes): *β-actin* and *GAPDH*. All samples were performed on three independent occasions. Quantification of gene expression was calculated by Microsoft Excel using the standards as described above or according to the 2^−△△C_T_ method (Schmittgen & Livak 2008).

### Quantification of *NLRP7* mRNA in oocytes and pre-implantation embryos

The oocytes and embryo samples used in the experiment included germinal vesicle (GV), 8 h, 16 h and metaphase II (MII) stage oocytes and parthenogenetic or *in vitro*-fertilized 2-cell, 4-cell, 8-cell, 16-cell, morula and blastocyst-stage embryos (*n* = 4 pools of 20 embryos). Procedures used for RNA isolation, cDNA synthesis, and quantitative real-time PCR analysis of mRNA abundance during *in vitro* oocyte maturation and pre-implantation embryonic development were conducted as described previously (Bettegowda *et al.* 2006, Tejomurtula *et al.* 2009

#### Western blotting

Western blot was performed as described previously (Pisani *et al.* 2010). In brief, proteins were extracted from ovine tissues following homogenization in RIPA lysis buffer and the tissue lysates were centrifuged at 12,000 × ***g*** for 15 min at 4°C. Protein concentration was measured using a bicinchoninic acid protein assay kit (Pierce). Equal amounts of protein were loaded per lane in SDS-PAGE and transferred onto a PVDF membrane (Immobilon; Millipore). The membrane was incubated with a blocking solution containing 5% skim milk in TTBS (10 mM Tris–HCl, pH 7.6, 137 mM NaCl, and 0.1% Tween 20), followed by incubation with the rabbit anti-NLRP7 antibody (N3C2, GeneTex, USA) at 1:1000 overnight at 4°C. After several rinses, membrane was incubated with goat-anti-rabbit IgG antibody (1:3000 dilution) labeled with horseradish peroxidase in blocking solution for 40 min at 37°C. Finally, the proteins were visualized with an enhanced chemiluminescence detection system according to the manufacturer’s instructions (GE Healthcare). *α*-actin (Bioss, 1:3000, Beijing, China) was used as a loading control.

### Immunohistochemistry

Ovaries were fixed in 4% paraformaldehyde overnight, dehydrated through gradient alcohol solutions and embedded in paraffin wax. Seven-micrometer sections were deparaffinized in xylene, rehydrated through gradient alcohol solutions and heated in 10 mM sodium citrate buffer (pH 6.0) with microwaves (10 min at high power) and cooled to room temperature for antigen retrieval. Immunostaining was performed using PV-9001IHC kits (ZSGB-Bio, China). In brief, sections were incubated with 1% BSA for 30 min at room temperature to block nonspecific binding, followed by incubation with the NLRP7 polyclonal antibody (GeneTex, N3C2, USA) (1:50 dilution of rabbit anti-NLRP7 antibody) in blocking solution for overnight at 4°C and with goat anti-rabbit secondary antibody (1:1500 dilution) for 30 min at room temperature. After several washes, the diaminobenzidine substrate solution was applied to the sections. The resultant sections were counterstained lightly with hematoxylin. Negative controls were performed via processing sections as above without the primary antibody. Previously published criteria were used to classify ovine follicles into specific developmental stages including the number of layers and appearance of granulosa cells (Fortune *et al.* 2003).

#### Immunofluorescence

All steps were performed at room temperature unless mentioned. The collected oocytes and embryos were fixed with 4% paraformaldehyde for at least 30 min, permeated for 20 min with 0.5% Triton X-100 in PBS, and all samples were incubated in the blocking solution (2% BSA, 2% skimmed milk powder, 0.15 mmol/L glycine and 0.05% Tween-20 in PBS) for 1 h, and then they were incubated with anti-NLRP7 (GeneTex, N3C2) antibody with a dilution of 1:200 for overnight at 4°C. After three times of washing with PBS containing 0.1% Tween-20 and 0.01% Triton X-100 for 5 min, the samples were incubated with FITC conjugated goat anti-rabbit second antibody (Abcam, dilution 1:500) for 2 h at 37°C. After three times of washing with PBS containing 0.1% Tween-20 and 0.01% Triton X-100 for 5 min, the DNA was stained with DAPI (C1005, Beyotime Institute of Biotechnology). Finally, the oocytes or embryos were mounted on glass slides and were observed with a fluorescent microscope.

### Partial CDS cloning and sequence analysis

A local Chinese breed, namely small-tailed Han sheep (genesis and species) was used in the present study. Ovaries were collected and total RNA was extracted from COCs using an RNA isolation kit (Invitrogen) according to the manufacturer’s instruction. Approximately 5 μg of total RNA were used as template for the synthesis of the first strand of cDNA by M-MLV reverse transcriptase (Promega). Based on the conserved regions of *Homo sapiens* (NM_001127255.1), *Bos taurus* (XM_002695413.4), *Capra hircus* (XM_018063100.1) and *Ovis aries* (XM_004015893.3) *NLRP7* mRNA sequences, a pair of primers was designed as the following: 5′- TAGTGCGATTGGGGTCTTG-3′ (forward) and 5′- CGCTATCTGGGATTGTTCTC-3′ (reverse). PCR was performed using 25 μL of PrimeSTAR GXL DNA polymerase in a final volume of 50 μL, containing 4 μL of dNTP mixture (2.5 mM each), 2 μL of primers (20 pmol/L), 2 μL of cDNA template (50 ng) and 5 μL of sterile water in 10 μL of 5× PrimeSTAR GXL Buffer, as instructed by the manufacturer (Takara). Amplification was performed in a DNA Thermal cycler (Mini Cycler Elite Thermal Cycler, MJ Research, Waltham, USA) under the following procedures: 35 cycles of denaturation at 98°C for 5 min, annealing at 60°C for 15 s, and extension at 68°Cfor 20 s. The final extension reaction was 5 min at 68°C. The PCR products were analyzed by electrophoresis on a 1% agarose gel, followed by purification and recovery using an agarose gel DNA purification and recovery kit (TaKaRa). The recovered fragments were cloned into the pMD18T vector (TaKaRa) and finally transformed into competent cells of *Escherichia coli* strain DH5α. White colonies were checked and the positive colonies were then sequenced (Sangon Biological Engineering and Technology and Service, Shanghai, China). Nucleotide sequences were analyzed using DNAMAN software. Multiple alignments of the NLRP7 proteins were performed using BLAST (www.ncbi.nlm.nih.gov/BLAST) and ClustalX (ftp://ftp-igbmc.u-strasbg.fr/pub/ClustalX) program. Phylogenetic analyses were conducted using neighbor-joining (NJ) methods using MEGA 7 (Kumar *et al.* 2016).

### **Oocyte collection and *in vitro* maturation** (**IVM)**

Ovine ovaries were collected from a commercial abattoir and transported to the laboratory in a thermos flask containing sterile saline solution supplemented with penicillin (100 IU/mL) and streptomycin (100 IU/mL) at 26–29°C within 2.5 h after slaughter. After three washes in fresh saline, COCs were recovered from the ovary with a blade in a culture dish of 90 mm diameter. COCs with a homogenous, evenly granulated ooplasm surrounded by at least three layers of compact cumulus cells were collected into Hepes-buffered tissue culture medium-199 (HTCM-199) supplemented with 0.1% polyvinyl alcohol (PVA), 0.04% of sodium bicarbonate, 25 IU/mL heparin, 0.065 g/L penicillin as well as 0.05 g/L streptomycin under a stereomicroscope for experiments. The tissue culture medium-199 (TCM-199) supplemented with sodium pyruvate (2.5 mM), l-glutamine (1.0 mM), penicillin (100 IU/mL), streptomycin (100 IU/mL), 10% fetal bovine serum (v/v, Gibco) and l-cysteine (0.1 mM) was used as the basal culture medium. Maturation medium was modified from basal culture medium by adding 100 ng/mL epidermal growth factor (EGF), 10 µg/mL follicle-stimulating hormone (FSH), 10 µg/mL luteinizing hormone (LH), and 1 µg/mL estradiol-17β. COCs were incubated for 24 h at 38.5°C in a humidified atmosphere under 5% CO_2_ in groups of 100 containing 800 µL maturation medium in four-well Petri dishes (Nunclon; Nalge Nunc, Roskilde, Denmark) covered with mineral oil.

### Parthenogenetic activation (PA) of oocytes

COCs were denuded with 0.1% hyaluronidase by pipetting and washed in maturation medium after 24 h of IVM. Parthenogenetic activation (PA) of ovine oocytes was performed as described previously (Quan *et al.* 2017), with some modification. Briefly, oocytes with a polar body were exposed for 5 min in 5 μM ionomycin in TCM 199 supplemented with 10% FBS followed by 4 h in 2 mM 6-dimethylaminopurine (6-DMAP) in TCM 199 supplemented with 10% FBS. The presumptive zygotes were then washed in modified synthetic oviduct fluid (mSOF) and transferred separately into pre-equilibrated 50 µL IVC droplets (10–15 zygotes/droplet) containing mSOF without serum and glucose for 2 days. Then, they were further treated with 10% fetal serum (10%) and glucose (1.5 mM) for another 5 days (sequential mSOF).

### *In vitro* fertilization (IVF)

IVM oocytes were fertilized in the synthetic oviductal fluid (SOF) medium with 2% ESS (estrous sheep serum) for 23 h at 38.5°C in an atmosphere of 5% CO_2_, 5% O_2_ and 90% N_2_ in four-well Petri dishes (Nunclon). Frozen-thawed sperm was selected by the swim-up method and was pelleted by centrifugation at 650 ***g*** for 10 min. The concentration of sperm in the resulting pellet was adjusted to 1×106 spermatozoa/mL. Thereafter, the presumptive zygotes were transferred in SOFaa medium with 0.3% bovine serum albumin (BSA) and cultured for 8 days (Bebbere *et al.* 2008). The first cleavage was observed between 24 and 26 h after the start of fertilization.

### RNA interference (RNAi) experiments

Three candidate shRNAs, *NLRP7*-2504shRNA, *NLRP7*-3122shRNA and *NLRP7*-3728shRNA, specially targeting ovine *NLRP7* gene (gene ID: 101102259) as well as a scrambled shRNA were designed by BLOCK-iT™ RNAi Designer (http://rnaidesigner.lifetechnologies.com/rnaiexpress). The sequences for them were listed in Table 2. These sequences were synthesized by Shanghai Sangon Biological Engineering Technology & Service Co. Ltd. (China) and were cloned into pZDonor expressing vector (Syngentech, China), which containing U6 promoter, shRNA, and termination sequences. The publicly available siRNA design algorithm (siRNA target finder, Ambion) was used to design siRNA species targeting the ORF of *NLRP7* mRNA in the *Ovis aries*. The candidate siRNA and shRNA species were interrogated by using the BLAST (Basic Local Alignment Search Tool) program to rule out homology to any other known genes (especially *NLRP2*) in the ovine EST and genomic database. Three distinct *NLRP7* siRNA species and one non-sense siRNA were synthesized by Shanghai GenePharma Co. Ltd. (China) and dissolved in nuclease free water. The sequences for them were listed in Table 3.

**Table 2:** *NLRP7* shRNA sequence.

shRNAs	Targets	shRNA sequence	TargetGene
pHS-ASR-LJ031	NLRP-7 Ovis aries 2504	ACCGCAGGTAGGAAAGAAGATATTCGAAAATATCTTCTTTCCTAC	*NLRP-7*
pHS-ASR-LJ032	NLRP-7 Ovis aries 3122	ACCGCTGTCTTCTGCTTTGATTGTCGAAACAATCAAAGCAGAAG	*NLRP-7*
pHS-ASR-LJ033	NLRP-7 Ovis aries 3728	ACCGCACTGAGATATTTCTCTTACCGAAGTAAGAGAAATATCTCA	*NLRP-7*
pHS-ASR-LJ006	Control	ACCGTAATTGTCAAATCAGAGTGCTTCAAGAGAAAGCACTCTGAT	Control

**Table 3:** NLRP7 siRNA sequence.

Targets	Sense (5′-3′)	Sense (5′-3′)
LOC101102259-Ovis-1464	GGAAGUUUACUGAAGAGAATT	UUCUCUUCAGUAAACUUCCTT
LOC101102259-Ovis-2614	GCUCUGUGCUCAGUUCAAATT	UUUGAACUGAGCACAGAGCTT
LOC101102259-Ovis-3602	GGACAAAUCUACCGUGGAATT	UUCCACGGUAGAUUUGUCCTT
Negative control	UUCUCCGAACGUGUCACGUTT	ACGUGACACGUUCGGAGAATT

Injection pipettes were made from sterile borosilicate glass with filament using a PN-30 micropipette puller (Narishige, Co., Tokyo, Japan), and angled at MF-900 with a microforge (Narishige, Co., Tokyo, Japan). Oocyte microinjections were performed on an Olympus IX71 inverted microscope (Olympus Co.) equipped with Transferman NK2 micromanipulators and CellTram Air/Oil pressure control systems (Eppendorf Co., Hamburg, Germany). The microinjection of sheep oocytes (*n* = 10–30 per treatment) with siRNAs and shRNA-plasmids was based on former studies (Paradis *et al.* 2005), with some modifications. Metaphase II stage oocytes were pretreated for 15 min in HB-TCM (Hepes buffered) supplemented with 7.5 mg/mL cytochalasin B before microinjection in droplets of prewarmed cycloheximide-supplemented HB-TCM under mineral oil. Three of the *NLRP7* siRNA duplexes as well as shRNAs plasmids were combined at a final concentration of 20 mM and 1 µg/µL, respectively, which was denoted as *NLRP7*-siRNA+shRNA group. Control groups consisted of oocytes injected with scrambled siRNA (nonspecific) mixed with nonsense shRNA plasmid (Nc-siRNA+shRNA group) and non-injected oocytes (non-injected group). Approximately 10 pl of mixture were injected into the cytoplasm of each oocyte using a microinjection pipette and a pneumatic PicoPump (World Precision Instruments, Stevenage, UK). After injection, the oocytes were maintained in HB-TCM medium at 38.5°C with a humidified atmosphere of 5% CO_2_ for 30 min followed by parthenogenetic activation or *in vitro* fertilization. Experiment was repeated four times.

After microinjection, parthenogenetic or IVF embryos at the 2-, 8-cell stage (*n* = 4 pools of 20 embryos per treatment) from *NLRP7*-siRNA + shRNA-injected group and control groups were collected to perform quantitative real-time RT-PCR for identification of *NLRP7* mRNA knockdown efficiency. The development of the uninjected or injected embryos (with *NLRP7* siRNA + shRNA or negative control siRNA + shRNA) was evaluated by recording the population of embryos 7 days after PA or IVF.

### Statistical analysis

All statistical analyses were performed using the IBM SPSS 22.0 software package (IBM corp. NY, USA, 2013). One-way ANOVA was used followed by a multiple pairwise comparison (Duncan’s test) to determine significant differences between the comparative groups. The results are expressed as the means ± s.e.m., and *P* values <0.05 were considered statistically significant. In addition, *t*-test was also applied to pairwise comparisons.

## Results

### Tissue distribution and intraovarian localization of ovine *NLRP7*

**Figure 1:**
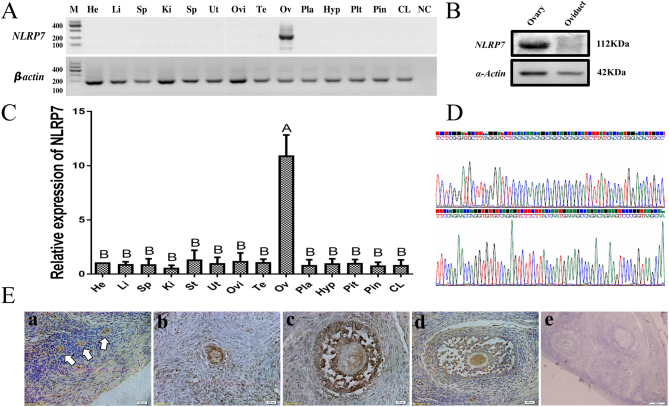
Tissue distribution and intraovarian localization of ovine *NLRP7*.(A) RT-PCR analysis of* NLRP7* mRNA transcripts in multiple ovine tissues. Samples of heart (He), liver (Li), spleen (Sp), kidney (Ki), stomach (St), uterus (Ut), oviduct (Ovi), testis (Te), ovary (Ov), placenta (Pla), hypothalamus (Hyp), pituitary (Pit), pineal gland (Pin) and corpus luteum (CL) were subjected to RT-PCR. M, Marker; NC, no PCR substrate as negative control. *β-actin* was used as a loading control. (B) Western blot of extracts obtained from sheep ovary and a control tissue (oviduct, randomly selected). Molecular masses (kDa) are indicated on the right. *α-actin* was used as an internal control.(C) Distribution of *NLRP7* mRNA in different ovine tissues demonstrated by quantitative real-time RT-PCR using *β-actin* and *GAPDH* as the reference genes; Experiments were repeated three times (*n* = 3) independently. Results have been normalized to the abundance in the heart (calibrator sample, randomly selected) and expressed as the mean ± s.e.m. Bars with different capital superscripts are significantly different (*P* < 0.01). (D) Sequencing results of *NLRP7* RT-PCR products.(E) Immunohistochemical localization of NLRP7 protein in the ovary sections. primordial follicles (a), primary follicle (b) and antral follicles (c, d,), e, negative control. Arrows are (a) indicators of primordial follicle. Scale bar = 200 µm.

To determine the tissue distribution of *NLRP7*, *NLRP7* mRNA expression in 14 different sheep tissues was analyzed using reverse transcription polymerase chain reaction (RT-PCR) with *NLRP7*-specific primers. As shown in Fig. 1A, *NLRP7* transcripts were strongly expressed in sheep ovary, with no detectable expression in the other tissues (including the heart, liver, spleen, kidney, stomach, uterus, oviduct, testis, placenta, hypothalamus, pituitary, pineal gland and corpus luteum). Similarly, quantitative real-time PCR analysis with two reference genes (*β-actin* and *GAPDH*) demonstrated that *NLRP7* mRNA was also mainly expressed in the ovary, with a neglected expression in other tissues (*P* < 0.01)(Fig. 1C). Western blot of NLRP7 protein in ovary and randomly picked control tissue (oviduct) shows the same results as RT-PCR (Fig. 1B). To validate the specificity of *NLRP7* primer, RT-PCR products were sequenced (Fig. 1D) and the sequences correspond to the NCBI database XM_004015893 as expected.

To further validate the expression of *NLRP7* in the ovary, immunohistochemistry staining was performed. Immunohistochemical localization of NLRP7 within adult ovary sections revealed that the protein was present in follicles regardless of their developmental stages (including primordial, primary and antral follicles) or the inner cell types (including oocytes, granulosa and theca cells) (Fig. 1E). It seems that the positive signal of NLRP7 in oocyte is weaker than that in granulosa/theca cells before the early antral stage (Fig. 1B, C and E). As the cavitation increases, the signal of NLRP7 in the oocytes becomes comparable to granulosa/theca cells (Fig. 1E-d). The ovarian stromal cells also showed a weak positive signal compared to the strong expression in follicles. Immunoreactivity was not detected in control tissue sections incubated in the absence of NLRP7 antibody.

### Expression pattern of NLRP7 protein in ovine COC, oocyte and parthenogenetic embryos

**Figure 2:**
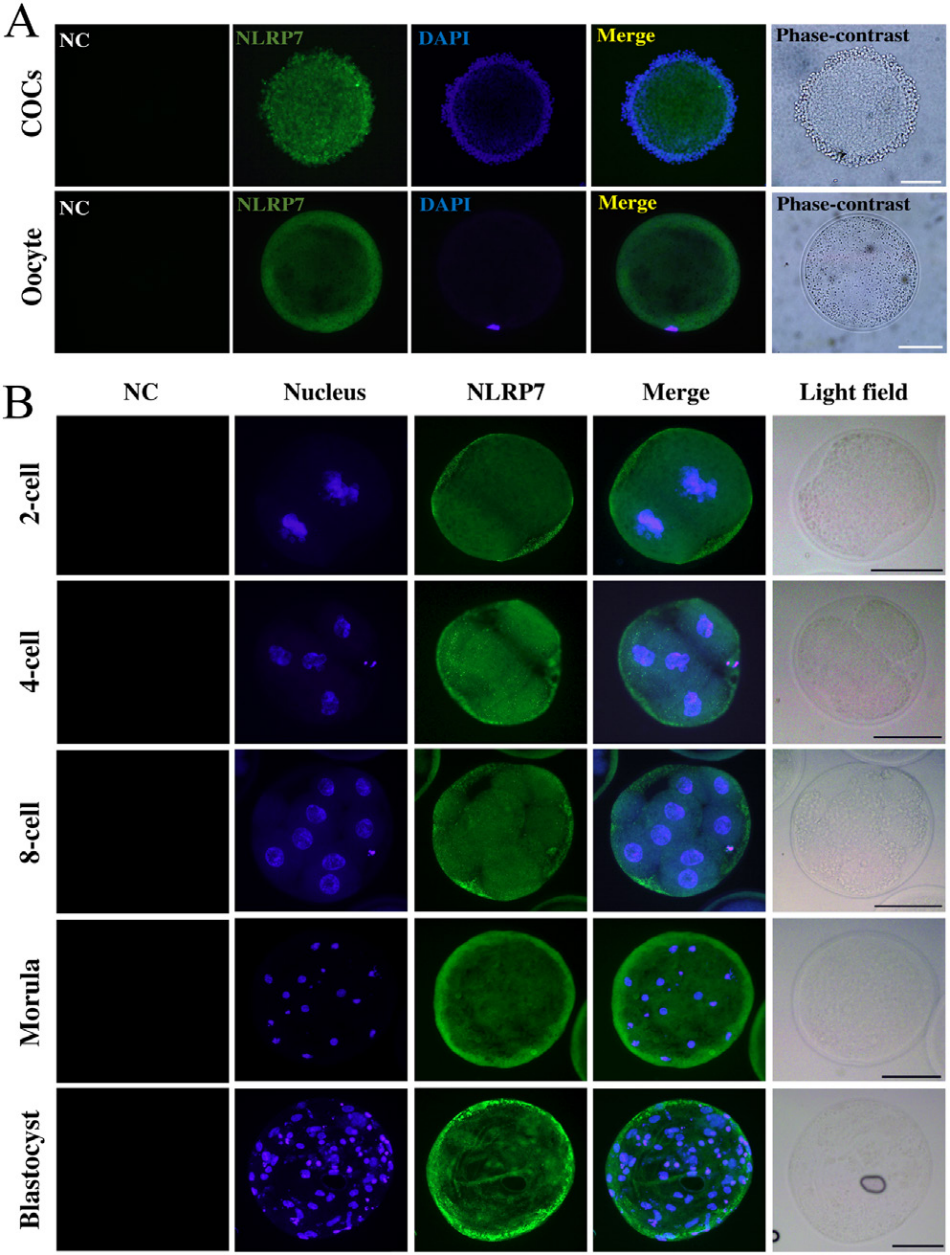
Expression pattern of NLRP7 protein in ovine COC, oocyte and parthenogenetic embryos. (A) Immunofluorescence with NLRP7 antibody (N3C2, Gene Tex) in COCs (cumulus oocyte complexes) and denuded oocyte.(B) Immunofluorescence of NLRP7 protein in parthenogenetic embryos. Scale bar = 50 µm. Omission of NLRP7 primary antibody shows no signal as negative control (NC). Each sample was counterstained with DAPI to visualize nucleus (blue).

Immunofluorescence staining confirmed that NLRP7 proteins were expressed in ovine COC, denuded oocyte (Fig. 2A) as well as in parthenogenetic embryos (two-cell, four-cell, eight-cell, morula and blastocyst) (Fig. 2B). In detail, NLRP7 was predominant at the outer cortical region in growing oocytes. After cleavage, NLRP7 distributed to the cytoplasm and was excluded from the cell-to-cell contact region until the morula stage. Furthermore, a clear nuclear signal is only evident starting from the blastocyst stage.

### Expression pattern of *NLRP7* mRNA in IVM oocytes and PA/IVF embryos

**Figure 3:**
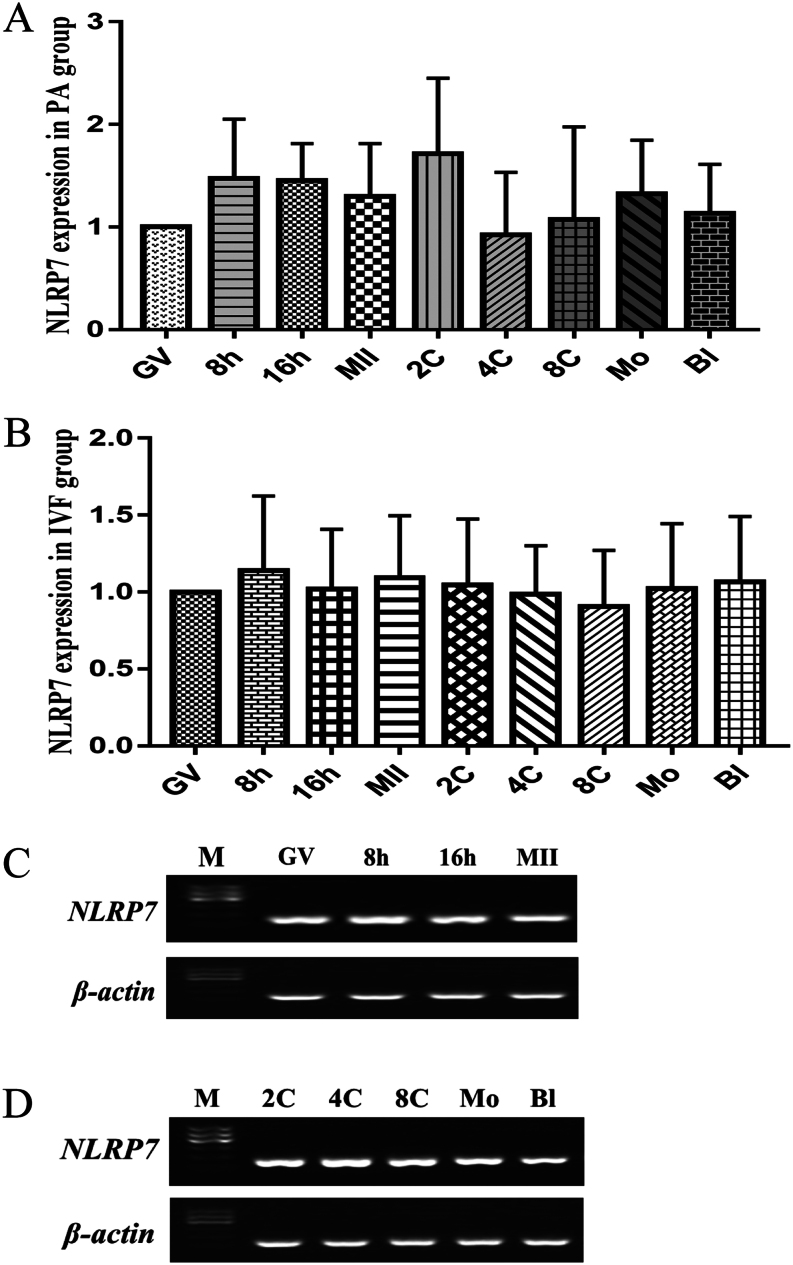
Expression pattern of *NLRP7* mRNA in IVM oocytes and PA/IVF embryos.(A) Relative abundance of NLRP7 transcripts in samples collected from GV (germinal vesicle), 8 h, 16 h, MII (Metaphase II) stage oocytes and PA (parthenogenetic activation) 2-cell (2C), 4-cell (4C), 8-cell (8C), morula (Mo) and blastocyst (Bl) stage embryos (*n* = 4 pools of 20 each). (B) Relative abundance of *NLRP7* transcripts in samples collected from GV (germinal vesicle), 8 h, 16 h, MII (Metaphase II) stage oocytes and IVF 2-cell (2C), 4-cell (4C), 8-cell (8C), morula (Mo) and blastocyst (Bl) stage embryos (*n* = 4 pools of 20 each). (C) RT-PCR of *NLRP7* in IVM oocytes.(D) RT-PCR of *NLRP7* in PA embryos. M, Marker. *β-actin* was used as an internal control.

For a better understanding of *NLRP7* mRNA abundance in oocyte maturation and *in vitro* pre-implantation embryos, its expression in *in vitro* maturation (IVM), parthenogenetic activation (PA) and *in vitro* fertilization (IVF) was evaluated by either real-time RT-PCR or RT-PCR. As shown in Fig. 3A, B, and C, the transcripts of *NLRP7* were abundant at GV (germinal vesicle), 8 h, 16 h and MII (Metaphase II) during oocyte maturation. Moreover, *NLRP7* mRNA also steadily expressed from two-cell to the blastocyst stage not only in PA but also in IVF embryos (Fig. 3A, B, and D). There were no significant differences at the stages between oocyte maturation and* in vitro* pre-implantation embryos development regarding the abundance of *NLRP7* mRNA expression (*P* > 0.05).

### Cloning and characterization of ovine* NLRP7* partial CDS

**Figure 4:**
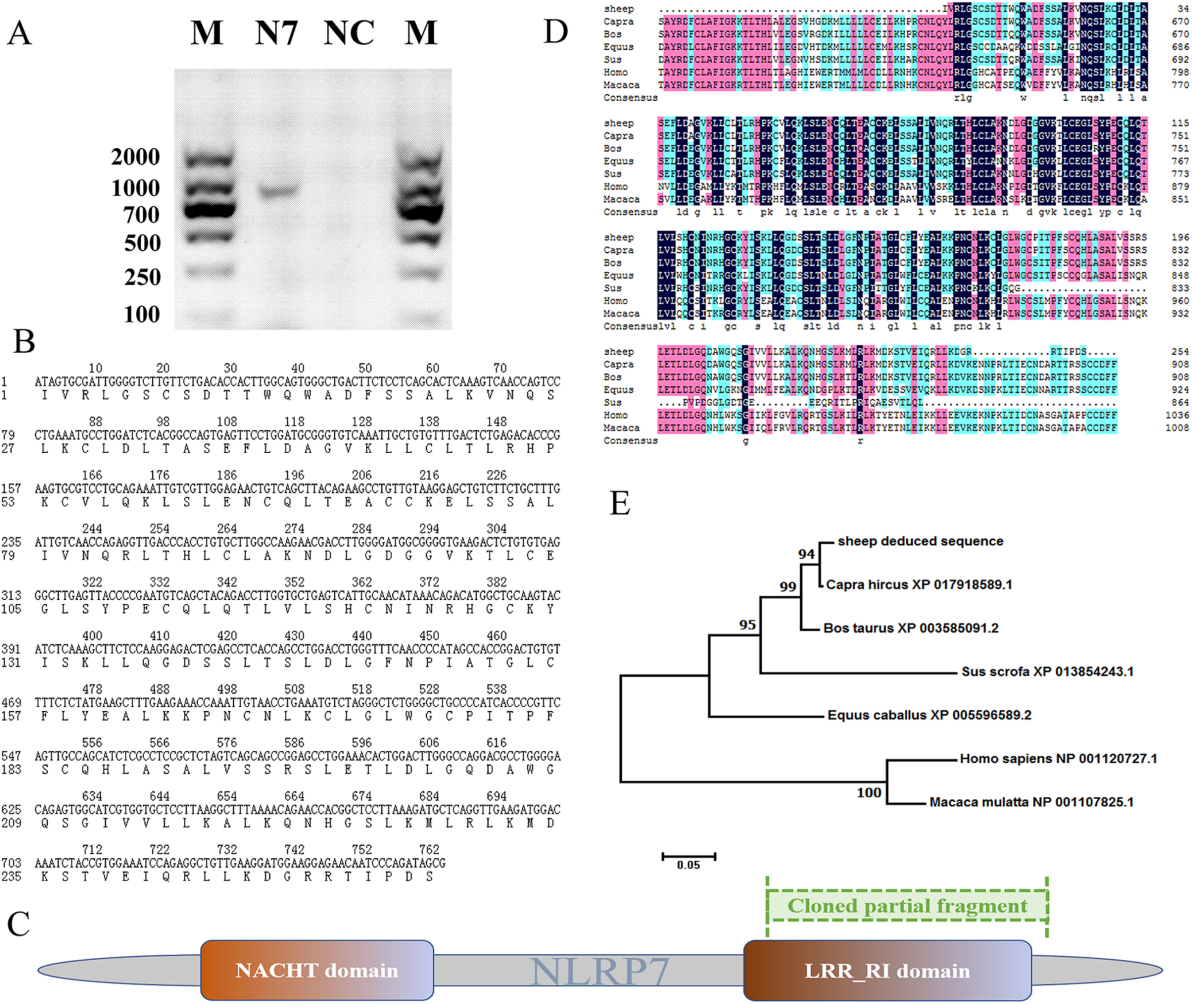
Cloning and characterization of ovine *NLRP7* partial CDS.(A) Reverse transcription-polymerase chain reaction (RT-PCR) amplification of the ovine *NLRP7* partial CDS. Lane N7, *NLRP7* amplicon; NC, negative control; M, 2000 bp DNA marker. The amplified ovine CDS is 763 bp.(B) Nucleotide and amino acid sequences of cloned *NLRP7* partial fragment of sheep. The deduced amino acid sequence (254 residues) is under the nucleotide sequence and numbered on the left. These sequence data have been submitted to the GenBank under an accession number MF197687.(C) Scheme of the cloned fragment (marked in green) compared to the full length of NLRP7 (marked in gray). NACHT and LRR_RI are two major domains of NLRP7. (D) Multiple alignments of NLRP7 amino acid sequence in different species including *Capra hircus*, *Bos Taurus*, *Homo sapiens*, *Macaca mulatta*, *Sus scrofa*, *Equus caballus*. The multiple alignments were produced using ClustalX, in which black color indicates positions that have a single, fully conserved residue (100% similarity) as opposed to pink (>75%), aqua (>50%), respectively. Dashes indicate gaps. (E) Phylogenetic tree of mammalian *NLRP7* homologs was conducted in MEGA7 using the Neighbor-Joining method, with the sum of branch length = 0.86954469. The percentage of replicate trees in which the associated taxa clustered together in the bootstrap test are shown next to the branches.

We have tried hard to clone the complete CDS of *NLRP7* (XM_004015893.3) gene by using different primers and programs, but failed to get the aiming amplicon. However, the partial coding sequence (CDS) was subsequently cloned using reverse transcript cDNA derived from ovine COCs. A 763-bp length sequence of sheep *NLRP7* was obtained (Fig. 4A), which containing 254 putative amino acid residues (Fig. 4B) and was submitted to GenBank (http://www.ncbi.nlm.nih.gov/genbank/; accession numbers: MF197687). The NLRP7 protein consists of two major domains: NACHT and LRR_RI. Figure 4C shows the scheme of the cloned fragment (marked in green) relative to the entire length of NLRP7 protein (marked in gray). The sequence obtained in sheep was analyzed with Blast and ClustalX program, which confirmed homology with the orthologous gene of public databases (Table 4 and Fig. 4D). The deduced protein sequence of NLRP7 shared 98% identity with *Ovis aries* (XP_004015942.2), 97% with *Capra hircus* (XP_017918589.1), 94% with *Bos taurus* (XP_002695459.2), 56% with *Homo sapiens* (NP_001120727.1), 57% with *Macaca mulatta* (NP_001107825.1), 82% with *Sus scrofa* (XP_020950638.1) and 76% with *Equus caballus* (XP_005596589.2) sequences. To determine the phylogenetic relationship of NLRP7 between sheep and other known or predicted sequences from other mammals, a phylogenetic tree was constructed using the deduced ovine NLRP7 protein sequence with MEGA 7 program. The analysis revealed that the highest homology was between sheep and goat and the lowest was between sheep and primates (Fig. 4E). In general, the amino acid sequence of NLRP7 protein is relatively well conserved among the mammals during evolution.

**Table 4:** Multi-alignment of NLRP7 with putative protein between different species.

Organism	Protein	Max score	Total score	Query cover	E value	Ident
Ovis aries	XP_004015942.2	494	764	98%	1e-174	98%
Capra hircus	XP_017918589.1	491	730	98%	9e-174	97%
Bos taurus	XP_002695459.2	473	624	98%	1e-166	94%
Homo sapiens	NP_001120727.1	269	608	99%	1e-87	56%
Macaca mulatta	NP_001107825.1	275	549	99%	4e-90	57%
Sus scrofa	XP_020950638.1	403	601	99%	1e-139	82%
Equus caballus	XP_005596589.2	372	480	98%	2e-127	76%

### Effect of *NLRP7* knockdown on *in vitro* pre-implantation embryo development

**Figure 5:**
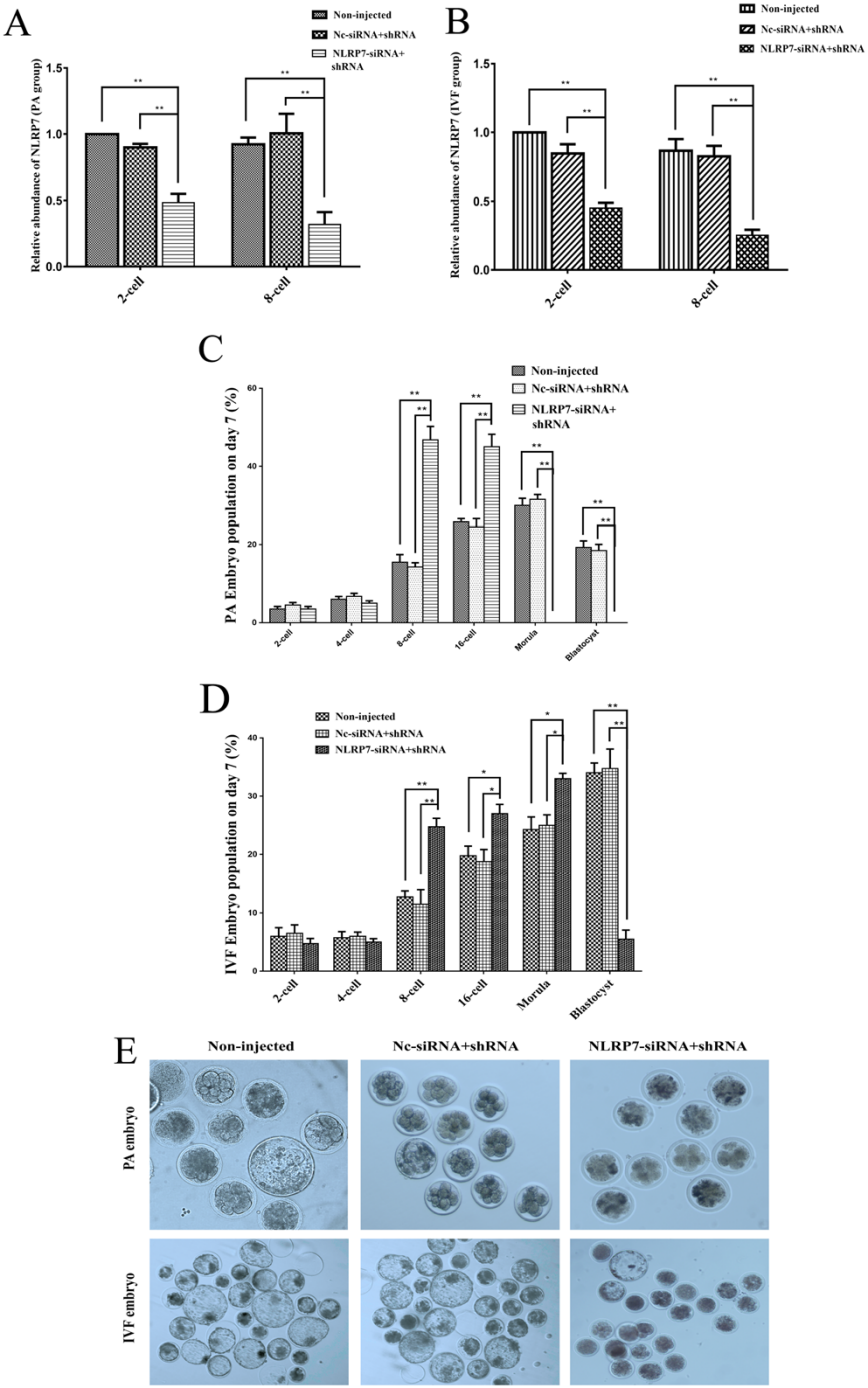
Effect of *NLRP7* knockdown on *in vitro* pre-implantation embryo development. Metaphase II oocytes were subjected to Non-injected control, NC-siRNA+shRNA injection or *NLRP7*-siRNA+shRNA injection (*n *= 10–30 embryos per treatment) following by PA or IVF. Microinjected embryos were cultured* in vitro* for 7 days. (A and B) Validation of *NLRP7* knockdown efficacy by quantitative real-time PCR in samples of 2-cell, 8-cell embryos in PA and IVF group respectively. Results were normalized to the 2-cell stage Non-injected control group and expressed as the mean ± s.e.m. Experiments were repeated four times (*n* = 4), **P < 0.01. (C and D) Percentage (mean ± s.e.m.) of PA and IVF embryos in three groups at different stages after culture for 7 days. Results were normalized to the 2-cell stage non-injected control group, **P < 0.01, *P < 0.05. (E) Morphology of PA and IVF embryos after 7 days of culture. The original magnification was 100×.

Considering the continuous expression of *NLRP7* in oocytes and embryos, we hypothesized that *NLRP7* is required for normal embryo development in ovine. To investigate the function of *NLRP7* in pre-implantation embryo development *in vitro*, RNAi to reduce *NLRP7* expression in PA and IVF embryos was carried out. Three *NLRP7* siRNA and shRNA species targeting different regions of the *NLRP7* gene were mixed into a cocktail to achieve potent and stable gene knockdown. Metaphase II oocytes were subjected to microinjection followed by PA or IVF. Microinjection of a cocktail of *NLRP7*-siRNA + shRNA reduced *NLRP7* mRNA levels by 51.6% (PA embryo)/54.9% (IVF embryo) at two-cell stage (*P* < 0.01) and 68.1% (PA embryo)/74.8% (IVF embryo) at eight-cell stage compared to non-injected control (*P* < 0.01)(Fig. 5A and B). To identify whether knockdown of *NLRP7* in ovine embryos has any effect on embryo development, PA/IVF embryos population on day 7 for *NLRP7*-siRNA+shRNA-injected vs non-injected and NC-siRNA + shRNA-injected group were determined. It turned out that the *NLRP7*-siRNA + shRNA injection jeopardized the normal development both in PA and IVF embryos (Fig. 5C, D, and E) compared to control group. These results clearly indicate that *NLRP7* is associated with ovine normal pre-implantation embryo development *in vitro*.

## Discussion

Previous studies reported that *NLRP7* was expressed in human immune cells lines of B, T, and monocytic cells (Messaed *et al.* 2011), as well as in variety of human tissues, including lung, spleen, thymus, and reproductive organs (testis and ovaries) (Kinoshita *et al.* 2005, Duéñez-Guzmán & Haig 2014). Furthermore, *NLRP7* expression was also detected in the human testicular seminomas cells and endometrial cancer tissues (Okada *et al.* 2004, Ohno *et al.* 2008). Current study indicated that *NLRP7* is mainly expressed in sheep ovaries and its expression pattern differed from that in human (Kinoshita *et al.* 2005). The expression pattern of *NLRP7* in sheep was similar to its paralog of *Nlrp2* in the mouse which was only detected in ovaries, but not in other tested tissues, even the testes (Peng *et al.* 2012). *NLRP7* expression was not restricted in the oocytes at various follicular stages but also in granulosa cells and theca cells. This expression pattern is also consistent with the observation on *Nlrp2* in mouse ovaries (Peng *et al.* 2012). Interestingly, immunohistochemistry staining showed that NLRP7 signal in oocyte was weaker than that in granulosa/theca cells before the early antral stage. This indicated that it might play some roles also in the autocrine/paracrine systems. The expression of* NLRP7* in ovine ovary indicates that its potential roles in the ovary function. Immunofluorescence analysis demonstrated a cytoplasm localization of NLRP7 protein close to the subcortex of oocytes, implying that this protein might be involved in cell cytoskeleton maintenance or signal transduction. Intracellular localization of NLRP7 protein in sheep oocytes was similar to the Nlrp2 protein in the mouse (Peng *et al.* 2012), as well as NLRP7 in the human (Akoury *et al.* 2015). In fact, among the human *NLRP* families, in addition to *NLRP5*, both *NLRP2* and *NLRP7* were considered as the subcortical maternal complex (SCMC) members (Mahadevan *et al.* 2017). Our study reveals that in pre-morula embryos, NLRP7 distributed into the cytoplasm and little signal was detected in the cell-to-cell contact region, which was very similar to the human NLRP7. In humans, at blastocyst stage, NLRP7 translocates from the nucleus to cytoplasm (Akoury *et al.* 2015). However, this appears not the case in sheep since there was a clear nuclear signal of NLRP7 protein in the blastocyst stage. This difference is presumably species specific. As a matter of fact, NACHT domain of NLRP7 possesses a nuclear localization signal (Slim & Wallace 2013, Reddy *et al.* 2016); thus, it is understandable that NLRP7 is located in the nucleus. Furthermore, the results also indicated that ovine NLRP7 blastocyst localization resembles *KHDC3L* in human (Akoury *et al.* 2015) and *Dnmt1o* in mice (Howell *et al.* 2001).

It was reported that the mouse *Nlrp2* mRNA accumulates during oogenesis and degrades (undetectable) after the two-cell stage in embryos, but the proteins persisted through to the blastocyst stage (Peng *et al.* 2012). However, in human, *NLRP4, 5, 8, 9, 11, 12, 13* and *14* were highly expressed in oocytes and then gradually decreased in embryos with a very low level in day 5 (D5) embryos, while *NLRP2* and *NLRP7* progressively decreased from oocytes to day 3 (D3) embryos, and then showed a sharp increase in D5 (Zhang *et al.* 2008). Our study showed that *NLRP7* mRNAs were abundant in oocyte maturation and PA or IVF embryos at different developmental stages, with no significant fluctuation, which was different from that observed in human pre-implantation embryos (Zhang *et al.* 2008).

In the current study, a 763 bp fragment (GenBank accession number: MF197687) of the *NLRP7* CDS from ovine COCs was cloned, which shared 99% identity (data not shown) to the predicted *Ovis aries NLRP7* mRNA in the NCBI database, with accession number XM_004015893. The deduced 254 amino acids peptide covered most of the LRR_RI domain (leucine-rich repeats, ribonuclease inhibitor-like), which was implicated in mediating autoregulation and protein–protein interaction as well as being typical of the NLRP family (Wagner *et al.* 2009). In accordance, the homology of the ovine NLRP7 protein with other mammalian species ranges from 97% identity in a 98% query cover with *Capra hircus*, to the 56 of 99% query cover with *Homo sapiens*, implying that the *NLRP7* gene is evolutionarily conserved among different species. In fact, many NLRP members possess conserved domains (Ye & Ting 2008). Moreover, the phylogenetic relationship of ovine *NLRP7* showed more affinity in ruminants than in primates, which is congruent with mammalian lineages topologies (Hedges *et al.* 2002, Reyes *et al.* 2004). On the other hand, to further study the structure and characteristics regarding ovine *NLRP7* gene in the ovaries, full length coding sequence including 3′- and 5′- ends remained to be investigated.

To study the function of *NLRP7* in ovine pre-implantation embryo development, RNAi was performed in Metaphase II stage oocytes followed by parthenogenetic activation or IVF. RNAi is a sequence-specific mRNA degradation induced by double-stranded RNA (dsRNA), which is usually induced either by delivering siRNA or shRNA into cells (Rao *et al.* 2009). RNAi became a favorable tool to study gene function also in mammalian oocytes and embryos. As a matter of fact, mouse oocytes were the first mammalian cell type where RNAi was conducted (Svoboda *et al.* 2000, Wianny & Zernicka-Goetz 2000). siRNA exhibits its effect immediately with transient gene silencing which is suitable for mouse embryos knockdown in which their developmental time was short. In contrast, shRNA is continuously synthesized by the host cell with much more durable effect, but it requires more time to transcribe from the vectors. In this study, the combination of siRNA and shRNA method was used to fulfill high potent and sustainable *NLRP7* gene knockdown. Our data demonstrated that *NLRP7* suppression in ovine embryos resulted in developmental impairment, indicating that *NLRP7* is required for normal embryo development *in vitro*. The results were similar to the report by Peng *et al.* (2012). *Nlrp2* knockdown in mouse leads to blockage at the two-cell stage both in fertilized and parthenogenetic embryos. Furthermore, others reported *Nlrp2*-deficient oocytes of mature adult mice showing defective parthenogenetic development (Kuchmiy *et al.* 2016). The similar phenomenon was also observed in *Nlrp4e*, *Nlrp5*, *Nlrp14*-deficient mice (Tong *et al.* 2000, Hamatani *et al.* 2004, Chang *et al.* 2013), as well as in rhesus macaque monkey (Wu *et al.* 2009) and sow (Peng *et al.* 2015).

The RNAi outcomes indicate that *NLRP7* mRNA knockdown does not preclude embryo development up to the 8–16-cells stage, while further development is arrested. These results can be interpreted in at least two ways: (1) *NLRP7* mRNA degradation is not sufficient to significantly decrease the cellular content of NLRP7 protein for several days, and thus, the effects of RNAi occur only when the endogenous protein is also degraded and cannot be replaced. (2) *NLRP7* functions in the late stage of pre-implantation embryo development (morula and blastocyst). This interpretation seems compatible with the translocation of *NLRP7* to a nuclear localization only at the blastocyst stage. Which is the case requires further investigations.

It was reported that maternal heterozygous *NLRP7* variant caused recurrent reproductive failure and imprinting disturbances in the offspring (Soellner *et al.* 2017)). Actually, *NLRP7* mutation impairs the normal protein biosynthesis, which is similar to its knockdown effect. In other ways, the *NLRP7* knockdown may disturb the natural DNA methylation status (Mahadevan *et al.* 2014) or influence the regular protein–protein interactions between the SCMC members (Mahadevan *et al.* 2017) in oocytes or embryos. Besides, previous studies have shown that *NLRP7* is important for the formation of inflammasome to protect against an immediate danger in the processing and maturation of pro-inflammatory cytokines, the interleukin, *IL-1β* and *IL-18* (Davis *et al.* 2011). Accordingly, knockdown of *NLRP7* probably weakened the embryo immunity. Certainly, to further investigate the* NLRP7* function in ovine, the over-expression, knock-out or other targeted inhibition experiments should be tested.

In conclusion, we identified that *NLRP7* was present in the ovine and its high expression was only detected in the ovary. The localization and expression patterns of *NLRP7* suggest its unique role in the female reproduction. Indeed, *NLRP7* knockdown is unfavorable for the pre-implantation embryo development *in vitro*. The functions of *NLRP7* in animal reproduction, particularly in husbandry important animals, deserve further exploration. The identification of *NLRP7* downstream signaling pathway to elucidate its molecular mechanisms is necessary and it is our future goal.

## Declaration of interest

The authors declare that there is no conflict of interest that could be perceived as prejudicing the impartiality of the research reported.

## Funding

This research was supported by funds from the National Science and Technology Major Project of China (2018ZX0800801B, 2016ZX08008003), National Natural Science Foundation of China (31830091), and the Beijing Dairy Industry Innovation Team (BAIC06-2017).
